# Effects of CEACAM1 in oral keratinocytes on HO-1 expression induced by Candida β-glucan particles

**DOI:** 10.1590/1678-7757-2022-0158

**Published:** 2022-11-04

**Authors:** Miyuki SAKUMA, Kouji OHTA, Shohei FUKADA, Misaki AKAGI, Hiroki KATO, Yoko ISHIDA, Takako NARUSE, Masaaki TAKECHI, Hideo SHIGEISHI, Hiromi NISHI, Tomonao AIKAWA

**Affiliations:** 1 Hiroshima University Graduate School of Biomedical and Health Sciences Department of Oral and Maxillofacial Surgery Minami-Ku Hiroshima Japan Hiroshima University, Graduate School of Biomedical and Health Sciences, Department of Oral and Maxillofacial Surgery, Minami-Ku, Hiroshima, Japan.; 2 Hiroshima University Graduate School of Biomedical and Health Sciences Program of Oral Health Sciences Minami-Ku Hiroshima Japan Hiroshima University, Graduate School of Biomedical and Health Sciences, Program of Oral Health Sciences, Department of Public Oral Health, Minami-Ku, Hiroshima, Japan.; 3 National Hospital Organization Kure Medical Center Chugoku Cancer Center Department of Dentistry, Oral and Maxillofacial Surgery Kure Japan National Hospital Organization Kure Medical Center and Chugoku Cancer Center, Department of Dentistry, Oral and Maxillofacial Surgery, Kure, Japan.; 4 Hiroshima University Hospital Department of General Dentistry Minami-Ku Hiroshima Japan Hiroshima University Hospital, Department of General Dentistry, Minami-Ku, Hiroshima, Japan.

**Keywords:** Candida albicans, CEACAM1, β-glucan-containing particles, HO-1, Oral keratinocytes

## Abstract

**Objective:**

Carcinoembryonic antigen-related cell adhesion molecule 1 (CEACAM1) is a member of the carcinoembryonic antigen family. Although its expression has been found in chronic oral inflammatory epithelium, this study aimed to know whether CEACAM1 in oral keratinocytes participates in host immune response against *Candida albicans* .

**Methodology:**

We investigated CEACAM1 expression in oral keratinocytes induced by *C. albicans* as well as by *Candida* cell wall component β-glucan particles (β-GPs). Furthermore, the effects of CEACAM1 on β-GPs-induced heme oxygenase-1 (HO-1) expression and its related signals were examined.

**Results:**

Fluorescence staining showed CEACAM1 expression in oral keratinocytes (RT7) cells, whereas quantitative reverse transcription (RT)-PCR indicated that both live and heat-killed *C. albicans* increased CEACAM1 mRNA expression in RT7 cells. Examinations using quantitative RT-PCR and western blotting indicated that CEACAM1 expression was also increased by β-GPs derived from *C. albicans* . Specific siRNA for CEACAM1 decreased HO-1 expression induced by β-GPs from *C. albicans* as well as the budding yeast microorganism *Saccharomyces cerevisiae* . Moreover, knockdown of CEACAM1 decreased β-GPs-induced ROS activity in the early phase and translocation of Nrf2 into the nucleus.

**Conclusion:**

CEACAM1 in oral keratinocytes may have a critical role in regulation of HO-1 for host immune defense during *Candida* infection.

## Introduction

Oral candidiasis is an oral mucosal infection caused by *Candida* species, and *C. albicans* is the most common associated-pathogen.^1^ *C. albicans* colonizes the surface of oral mucosa, where it adheres, and invades oral keratinocytes, leading to damage during oral infection.^2^ On the other hand, oral keratinocytes in the oral mucosal membrane are the first line of defense against *C. albicans* in innate immune systems. Oral keratinocytes can produce inflammatory cytokines, chemokines, and anti-microbial peptides against live *Candida* ,^3 , 4 , 5^ and increase the expression of various genes related to inflammatory and stress responses by exposure to heat-killed *C. albicans.* ^6^ These reports also indicated that oral keratinocytes can recognize *Candida* cell wall components, as well as live *Candida* , and an induce immune response against *Candida* infection.

Carcinoembryonic antigen-related cell adhesion molecule 1 (CEACAM1) is a member of the carcinoembryonic antigen (CEA) family and associates with various signal transduction processes related to cell activation, proliferation, differentiation, and apoptosis.^7^ This molecule expression, induced by inflammatory cytokines and specific bacteria,^9 , 10^ takes place on the surface of epithelial and endothelial cells, as well as various immune cell types, such as neutrophils, monocytes, dendritic cells, NK cells, T cells, and B cells.^8^ Furthermore, CEACAM1 modulates immune response, which regulates inflammatory signaling and cytokine genes in various cell types.^11 , 12^ In the oral cavity, cases of chronic inflamed oral epithelium present CEACAM 1 expression, such as periodontitis and lichen planus.^13 , 14^ However, if CEACAM1 in oral keratinocytes participates in host defense systems against *C. albicans* remains unknown.

The β-glucan, a major cell wall component of *C. albicans* as well as the budding yeast *Saccharomyces cerevisiae* , constitutes the inner layer of mannan/mannoprotein, which is in the outer layer of the *Candida* cell wall.^15^ This component has an important function as an immune modulator, since it can activate immune response in immune cells, such as monocytes and macrophages.^16^ Previously, we demonstrated that β-glucan particles (β-GPs) derived from *C. albicans* enhanced expression of heme oxygenase-1 (HO-1), a stress-inducible gene, in oral keratinocytes, which contributes to host defense against stress caused by *Candida* infection.^6^ However, CEACAM1 functions as a cellular receptor, and interacts with several bacterial pathogens as well as fungi.^17 - 21^ A recent report noted that *C. albicans* directly interacts with CEACAM1 by the N-terminal IgV-like domain and β-glucan has been found on the surface of human oral epithelium after invasion by *Candida* .^6^ Therefore, β-glucan exposed on the surface of *C. albicans* may be associated with CEACAM1-mediated immune response in oral keratinocytes.

Based on the hypothesis that CEACAM1 in oral keratinocytes participates in host immune response against *C. albicans* , we investigated CEACAM1 expression in oral keratinocytes induced by *C. albicans* and the cell wall component β-GPs. Furthermore, we examined the effects of CEACAM1 on the intercellular signaling pathway involved in β-GP-induced HO-1 expression.

## Methodology

### Reagents

The following antibodies used for immunoblotting were obtained: anti-CEACAM1 (MAB22441) (R&D Systems, Minneapolis, MN, USA); anti-CEACAM1 (ab182622) (Abcam, Cambridge, MA, USA); anti-Nrf2 (BS1258) (Bioworld Technology, St. Louis Park, MN, USA); anti-glyceraldehyde-3-phosphate dehydrogenase (GAPDH) (MAB374) (Millipore Corporate Headquarters, Billerica, MA, USA); and anti-lamin B (E13490) (Spring Bioscience, Pleasanton, CA, USA). Neutralizing antibodies used were anti-CEACAM IgG (Novus Biologicals, Centennial, CO, USA) and mouse IgG (R&D Systems). Secondary antibodies employed for labeling were a horseradish peroxidase (HRP)-conjugated antibody from GE Healthcare Life-Sciences (Tokyo, Japan) and an Alexa Fluor secondary antibody 488 goat anti-mouse antibody (Thermo Fisher Scientific Life Technology Japan, Tokyo, Japan). Yeast whole β-glucan particulates (WGPs) extracted from *S. cerevisiae* were purchased from Invivogen (San Diego, CA, USA).

### Cell lines

The RT7, an immortalized human oral keratinocyte cell line, was established by transfection of hTERT and E7, as previously described,^22^ then those cells were cultured in keratinocyte growth medium (KGM), supplemented with human epidermal growth factor, insulin, hydrocortisone, calcium, bovine pituitary extract, and gentamicin sulfate amphotericin-B (Lonza, Walkersville, MD).

### Microorganisms and growth conditions

*C. albicans* IFO1385 is the standard strain used in related study,^23^ and was generously provided by the Department of Bacteriology, Hiroshima University Graduate School of Biomedical Sciences. After growth in Sabouraud broth medium at 37°C overnight, the microorganisms were washed twice with phosphate buffered saline (PBS) and then heat-killed at 60°C for 30 minutes.^24^

### β-glucan-containing particles derived from *C. albicans*

*C. albicans* β-GPs were obtained using a hot alkali and acid method, as previously described.^25^ *C. albicans* IFO1385 was cultured in 1200 mL of Sabouraud’s broth medium, then washed with PBS and suspended in 1% (wt/v) NaOH at 100°C for 24 h, and later the supernatant for the alkali-soluble fraction was collected. The insoluble residue was treated with 0.5 M acetic acid at 80°C for 24 h and subjected to repeated washings, then neutralized with PBS (pH 7.4), which the pellet was later lyophilized and stored at 4°C.

### Immunocytochemistry

Cells were seeded into two-well chamber slides (Matsunami Glass, Osaka, Japan) and fixed in 4% paraformaldehyde in PBS for 15 minutes, followed by permeabilization with 0.2% Triton X-100 in PBS for 5 minutes and incubation overnight at 4°C with the primary antibody (mouse IgG at 1:200 dilution, anti-CEACAM1 at 1:100 dilution) in PBS containing 5% BSA. Next, cells were washed and incubated with diluted Alexa Fluor secondary antibody (1:500 dilution) for 1 h. Vectashield anti-fade medium containing DAPI (Vector Laboratories, Burlingame, CA, USA) was used to mount the cells. Fluorescent and phase contrast images were acquired with a BZ-9000 microscope (KEYENCE, Osaka, Japan).

### RNA extraction

RT7 cells were seeded into cell culture plates and cultured until reaching 70-80% confluence. After washing the plates with PBS and transferring to antibiotics-free medium, they were incubated with live *C. albicans* (10^5^ CFU/mL), heat-killed *C. albicans* (10^8^ CFU/mL), or β-GPs (200 μg/mL) for 12 h. Those concentrations were previously determined to not significantly increase lactate dehydrogenase release by the cells relative to the non-infected control cultures using a cytotoxicity detection kit (Roche Applied Science)^6^. RNA from the cultures was extracted using a RNeasy Mini Kit (Qiagen, Hilden, Germany). Single-stranded cDNA used for a polymerase chain reaction (PCR) template was synthesized using a First Strand cDNA Synthesis Kit (Amersham Biosciences, Uppsala, Sweden), then applied in reverse transcription (RT)-PCR and real-time PCR assays.

### RT-PCR and real-time PCR

Synthesized cDNA was used for quantitative PCR analysis with the following oligonucleotide primers: CEACAM1, TGACACAGGACCCTATGAGT and ACTGTGCAGGTGGGTTAGAG; HO1, TCCGATGGGTCCTTACACTC and ATTGCCTGGATGTGCTTTTC; b-actin, TCACCCAC-ACTGTGCCCATCTACGA and CAGCGGAACCGCTCATTG-CCAATGG (Hokkaido System Science, Hokkaido, Japan). RT-PCR was performed using an RT-PCR High Plus system (Toyobo, Osaka, Japan), according to the manufacturer’s instructions. The RT-PCR conditions for CEACAM1 were 1× (94°C, 5 minutes), 35× (94°C, 1 minute; 60°C, 1 minute; 72°C, 1 minute), and 1× (72°C, 10 minutes), while those for β-actin were 1× (94°C, 5 minutes), 35× (94°C, 1 minute; 60°C, 1 minute; 72°C, 1 minute), and 1× (72°C, 10 minutes). The products were analyzed on 2% agarose gels, containing Gotaq Green Master Mix (Promega Corporation). Quantitative PCR analysis was performed using a CFX Connect Real-Time PCR Detection System (Bio-Rad Laboratories, Inc., Hercules, CA, USA) and THUNDERBIRD^TM^ SYBR qPCR Mix (Toyobo) for 40 cycles at 95°C for 15 seconds, and then at 60°C for 60 seconds. Relative quantification of mRNA levels, noted for the samples, was performed according to User Bulletin #2 (Applied Biosystems). The mRNA level for each gene in the samples was normalized according to β-actin mRNA, then calculated using the 2^Ct^ (-delta CT) method. Compared to the control, values are shown as fold increase, with results from at least three independent experiments presented as the mean standard deviation.

### Preparation of whole cell extracts, and nuclear fractions

Cell cultures were washed with ice-cold PBS, then subjected to lysis with sodium dodecyl sulfate (SDS) sample buffer using a Mammalian Cell Lysis Kit (Sigma-Aldrich, St. Louis, MO, USA) to yield whole cell extracts. Nuclear fractions were extracted from the cell cultures with a nuclear kit (Cayman Chemical Company, Ann Arbor, MI, USA). In a preliminary study, western blotting with cell extracts was performed using the nuclear extraction kits with lamin B as the internal control. The results confirmed that the cultured cells could be divided into those fractions.^6^

### Western blotting

Proteins from each sample were separated on 10% SDS-polyacrylamide gels and left for 1 h at 100 V, then transferred to polyvinylidene fluoride membranes (Amersham Biosciences) for 1 h at 90 V. After blocking for 1 h at room temperature with 5% BSA in PBS, the membrane was incubated with the primary antibody (1:1000 dilution) at an appropriate dilution at 4°C overnight. Immunoblots were labeled with an HRP-conjugated secondary antibody (1:1000 dilution) at an appropriate dilution for 1 h at room temperature and developed using an ECL Advance Western blotting Detection Kit (GE Healthcare Life Science, Tokyo, Japan). Image data were analyzed with an LAS 4000 mini-imaging system (Fuji Film, Tokyo, Japan). The ImageJ software package, version 1.47 (NIH), was used to analyze the intensities of the western blot bands. The ratio of the band density of each target protein to that of GAPDH was calculated.

### Small interfering RNA

Stealth small interfering RNA (siRNA) complexes for CEACAM1 #1 and #2 were designed by and purchased from Japan Bio Services Co., LTD. The siRNA sequences were: CEACAM1 #1, 5’-CCA UUG CUG GCA UUG UGA UUG GAG U-3’ and 5’-ACU CCA AUC ACA AUG CCA GCA AUG G-3’; CEACAM1 #2, 5’- CCA GUC ACC UUG AAU GUC ACC UAU G’ and 5’-CAU AGG UGA CAU UCA AGG UGA CUG G-3’. Negative control siRNA (Stealth RNAi™ siRNA Negative Control) was purchased from Invitrogen.

### Determination of intracellular ROS production

The fluorescent probe DCF-DA (2’, 7’-dichlorodihydrofluorescein diacetate) (Sigma-Aldrich) was used to determine the intracellular accumulation of ROS.^26^ RT7 cells were washed with PBS and incubated in medium containing 10 μM DCF-DA at 37°C for 45 minutes, then the medium was removed and replaced with fresh containing. Next, the cells were incubated with various concentrations of β-GPs and washed three times with PBS. Using a fluorescence microplate reader (Appliskan Thermo Scientific, Waltham, MA, USA), fluorescent intensity was determined and presented as relative fluorescence units, with an excitation wavelength of 485 nm and emission wavelength of 530 nm.

### Statistical analysis

Results were analyzed using one-way analysis of variance (ANOVA), followed by a Bonferroni/Dunn’s post hoc test for multiple comparisons. All values are presented as the mean standard deviation of at least three independent experiments.

## Results

### Effects of *C. albicans* and β-GPs on CEACAM1 expression in oral keratinocytes

**Figure 1:**
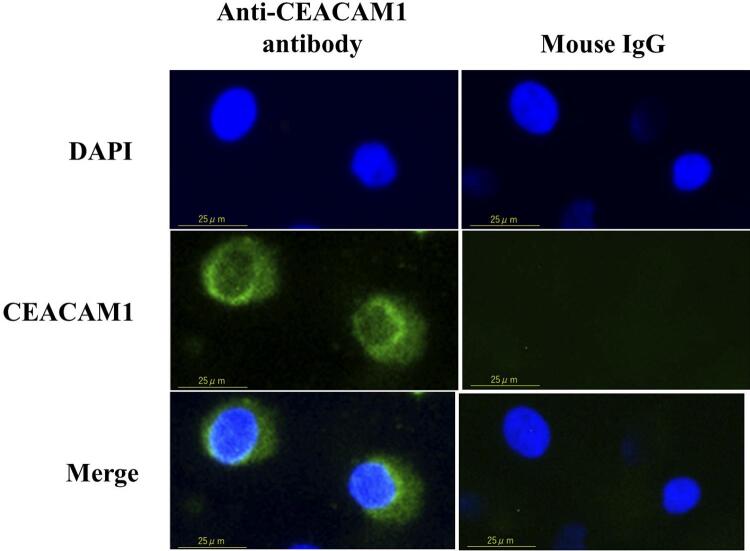
Expression of CEACAM1 in oral keratinocytes and fibroblasts Localization of CEACAM1 expression in RT7 cells. Cells were stained with anti-CEACAM1 or mouse IgG as a negative control along with Alexa Fluor^®^ 488 conjugated mouse IgG, and nuclei were counter-stained with DAPI (blue). Green staining indicates CEACAM1. Each experiment was performed at least three times, with representative results shown.

**Figure 2:**
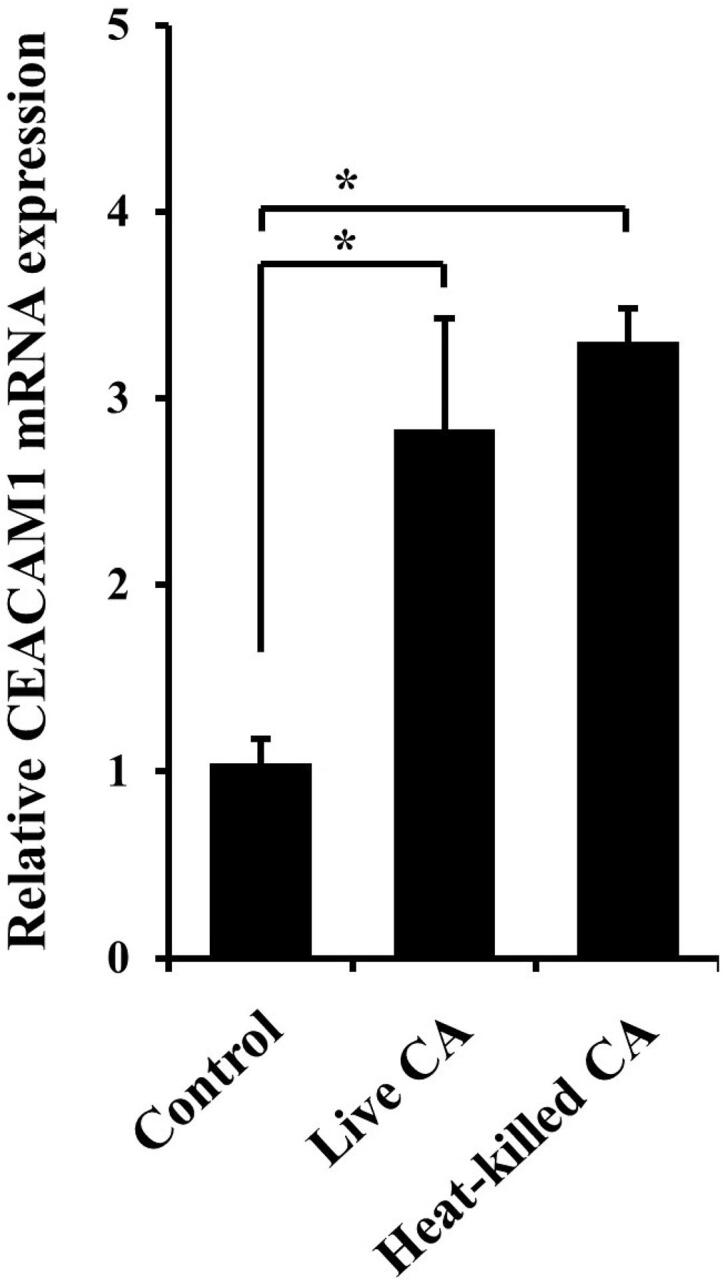
Effects of live and heat-killed *C. albicans* on CEACAM1 mRNA expression in RT7 cells. Cells were incubated with live (105/mL) or heat-killed (108/mL) *C. albicans* (CA) for 12 hours. Gene mRNA levels are presented as relative to that of β-actin. Values are shown as fold increase as compared to non-treated cells and presented as the mean ± SD of three independent experiments. *Significantly different from non-treated control (Bonferroni/Dunn's multiple comparison test: p<0.05).

**Figure 3:**
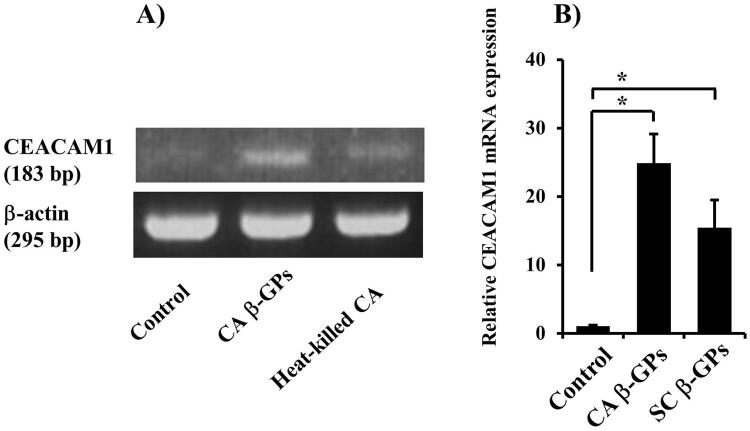
Effects of fungal β-GPs on CEACAM1 mRNA expression in RT7 cells (A) Cells were incubated with *C. albicans* β-glucan-containing particles (CA β-GPs) (200 μg/mL) or heat-killed (108/mL) *C. albicans* (CA) for 12 hours. Gene mRNA levels are presented as relative to that of β-actin. Then, RT-PCR assays were performed for CEACAM1 and β-actin. The experiments were performed at least three times, with representative results shown. (B) Cells were incubated with CA β-GPs (200 μg/mL) or *S. cerevisiae* β-GPs (SC β-GPs) (200 μg/mL) for 12 hours. Gene mRNA levels are presented as relative to that of β-actin. Values are shown as fold increase as compared with non-treated cells and presented as the mean ± SD of three independent experiments. *Significantly different from non-treated control (Bonferroni/Dunn's multiple comparison test: p<0.05).

**Figure 4:**
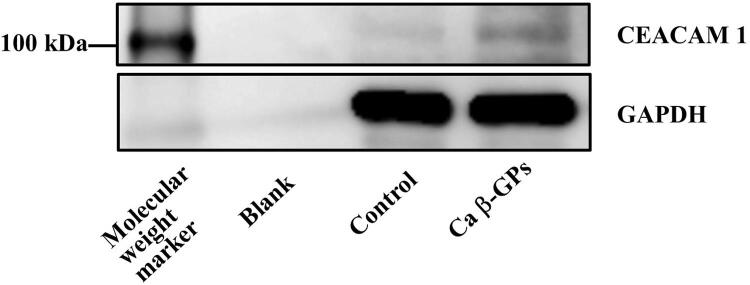
Effects of *C. albicans* β-GPs on CEACAM1 protein expression in RT7 cells Cells were incubated with *C. albicans* β-glucan-containing particles (CA β-GPs) (200 μg/mL) for 24 hours. Cell extracts were subjected to SDS-PAGE and western blotting with antibodies against CEACAM1 and GAPDH. The experiments were performed at least three times, with representative results shown.

To investigate the effects of *C. albicans* on CEACAM expression in oral keratinocytes, we first determined if oral keratinocytes express CEACAM1, showed by the fluorescence staining ( Figure 1 ). Next, we examined the effects of live and heat-killed *C. albicans* on CEACAM1 expression in RT7 cells and oral keratinocytes, which both lived and heat-killed *C. albicans* , increasing CEACAM1 mRNA expression in those cells ( Figure 2 ). Furthermore, we examined the ability of β-GPs, a *C. albicans* cell wall component, to affect CEACAM1 expression, and RT-PCR analysis showed that *C. albicans* β -GPs, as well as heat-killed *C. albicans* , increased that expression ( Figure 3A ). Composed of a β-glucan layer covalently linked to a variety of cell surface mannoproteins,^27^ *C. albicans* and the budding yeast *S. cerevisiae* share cell wall structure similarities. *S. cerevisiae* and *C. albicans* β-GPs ( Figure 3B ) increased CEACAM1 expression, whereas western blotting results indicated that *C. albicans* β-GPs increased CEACAM1 protein expression, approximately 120 kDa, also noted in a previous report^28^( Figure 4 ).

### Effects of CEACAM1 on β-GPs-induced HO-1 expression

**Figure 5:**
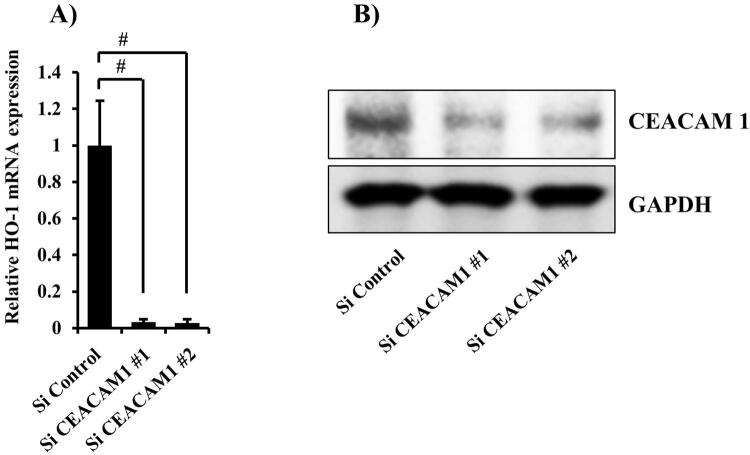
Knockdown of CEACAM1 expression by siRNA (A) Cells were transfected with CEACAM1 siRNA for 48 hours. Gene mRNA levels are presented relative to that of β-actin. Values are shown as fold increase as compared with non-treated cells and presented as the mean±SD of three independent experiments. #Significantly different from Si control-transfected cells (Bonferroni/Dunn's multiple comparison test: p<0.05). (B) Cells were transfected with CEACAM1 siRNA for 48 hours, after which cell extracts were subjected to SDS-PAGE. Western blotting analysis with antibodies against anti-CEACAM1 and GAPDH was performed. The experiments were performed at least three times, with representative results shown

**Figure 6:**
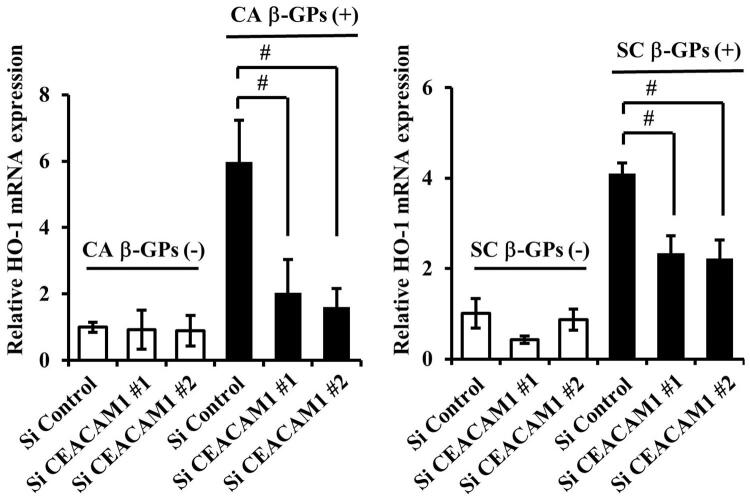
Effects of CEACAM1 siRNA on β-GPs-induced HO-1 in RT7 cells Cells were transiently transfected with siRNA for CEACAM1 for 48 hours, then exposed to *C. albicans* β-glucan-containing particles (CA β-GPs) or *S. cerevisiae* β-GPs (SC β-GPs) (200 μg/mL) for 12 hours. Gene mRNA levels are presented as relative to that of β-actin. Values are shown as fold increase as compared to control siRNA-transfected cells and presented as the mean ± SD of least three independent experiments. #Significant decrease as compared with the control (Bonferroni/Dunn's multiple comparison test: p<0.05).

**Figure 7:**
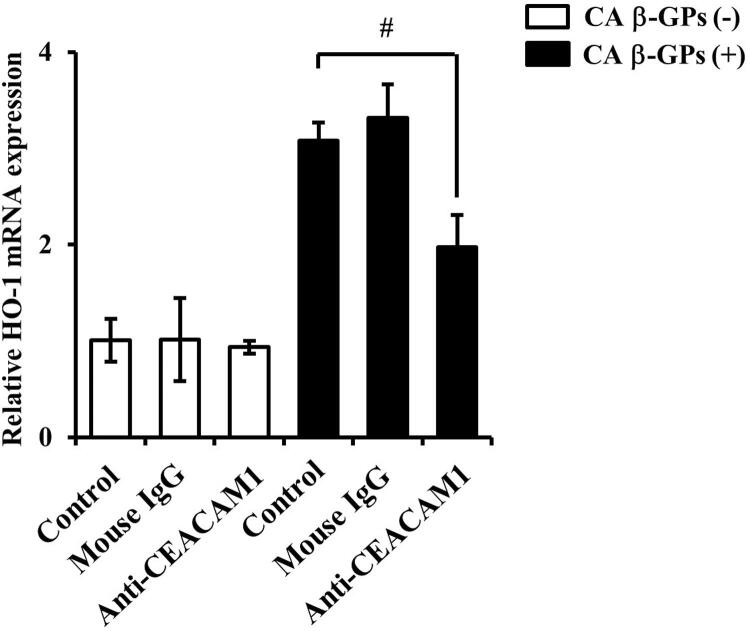
Effects of CEACAM1 neutralizing antibody on β-GPs-induced HO-1 mRNA expression in RT7 cells Cells were pre-incubated with the neutralizing antibody for CEACAM1 and mouse IgG as the negative control (each 10 μg/mL) for 1 hour, then exposed to *C. albicans* β-glucan-containing particles (CA β-GPs) (200 μg/mL) for 12 hours. HO-1 mRNA levels are shown as relative to that of β-actin. Values are shown as fold increase as compared to the control cells and presented as the mean ± SD of three independent experiments. #Significant decrease as compared with *C. albicans* β-GPs alone (Bonferroni/Dunn's multiple comparison test: p<0.05).

In our previous study, we found that β-GPs from both *C. albicans* and *S. cerevisiae* enhance expression of the stress-inducible gene HO-1 in oral keratinocytes.^6^ To determine if β-GPs-induced HO-1 expression is associated with CEACAM1 expression, we examined knockdown of CEACAM1, mediated by two different siRNAs ( Figure 5A , B ), which showed that knockdown by each siRNA decreased HO-1 expression induced by β-GPs from both *C. albicans* and *S. cerevisiae* ( Figure 6A , B ). Furthermore, the neutralizing antibody for CEACAM1 also decreased β-GPs-induced HO-1 expression ( Figure 7 ). Therefore, β-GPs-induced HO-1 and CEACAM1expressions are associated.

### Effects of CEACAM1 on signaling mediated by β-GPs-induced HO-1 expression

**Figure 8:**
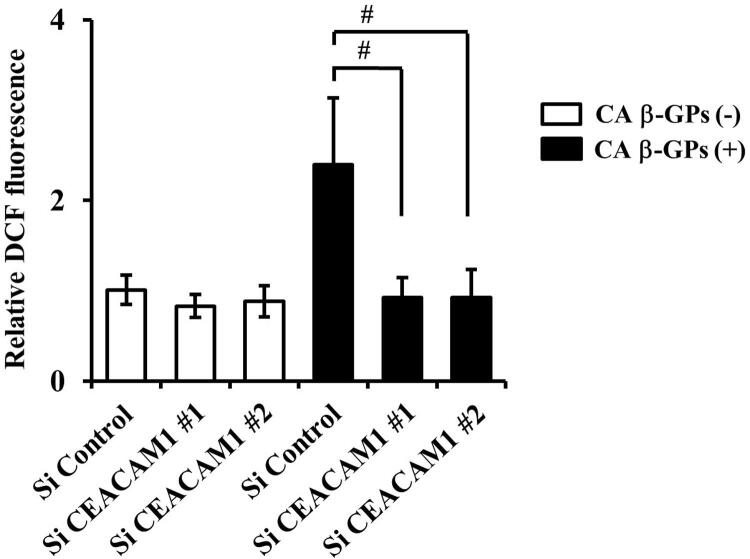
Effects of knockdown of CEACAM1 on β-GPs-induced reactive oxygen species in RT7 cells Cells were transfected with CEACAM1 siRNA for 48 hours, then labelled with DCF-DA for 30 minutes and stimulated with *C. albicans* β-glucan-containing particles (CA β-GPs) (200 μg/mL) for 1 hour. Fluorescence intensity was then determined. Values are shown as fold increase as compared with the intensity of Si control cells and presented as the mean ± SD of five independent experiments. #Significantly different from Si control-transfected cells stimulated with C. albicans β-GPs alone (Bonferroni/ Dunn's multiple comparison test, p<0.05).

**Figure 9:**
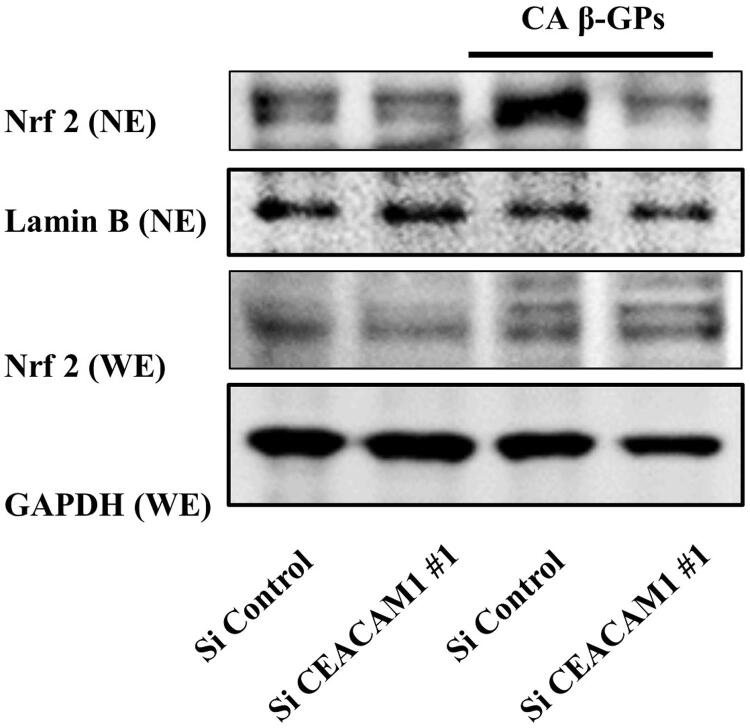
Effects of knockdown of CEACAM1 on β-GPs-induced translocation of Nrf2 into nucleus in RT7 cells Cells were transfected with siRNA for CEACAM1 siRNA for 48 hours, then stimulated with *C. albicans* β-glucan-containing particles (CA β-GPs) (200 μg/mL) for 4 hours. Whole cell and nuclear extractions were prepared, then subjected to western blot analysis with the Nrf2 antibody. LaminB and GAPDH were used as marker proteins for the nuclear and whole cell fractions, respectively. The experiments were performed at least three times, with representative results shown.

Oxidative stress is caused by elevation of reactive oxygen species (ROS). ^29^ We previously demonstrated that β-GPs increased HO-1 expression by enhancement of intercellular ROS.^6^ Another study found ROS-mediated signaling activating nuclear factor erythroid 2-related factor 2 (Nrf2), a recently revealed regulator of cellular resistance to oxidants, which is translocated into the nucleus and increases HO-1 expression.^30^ We examined if signaling related to β-GPs-induced HO-1 expression mediates CEACAM1. In the early phase, the knockdown of CEACAM1 decreased β-GPs-induced intracellular ROS production by its specific siRNA ( Figure 8 ). Furthermore, Nrf2 expression in transfected cells with CEACAM1 siRNA was high in the nucleus, as compared to control siRNA cells, suggesting a decrease in translocation of Nrf2 into the nucleus by knockdown of CEACAM1 ( Figure 9 ). Therefore, CEACAM1 is associated with regulation of oxidative stress-mediated signaling related to β-GPs-induced HO-1 expression.

## Discussion

CEACAM1 is found in a variety of human organs, including gastric, colon, lung, breast, endometrium, prostate, and gall bladder tissues. In oral tissues, CEACAM1 was detected in sulcular epithelium sites affected by periodontitis and junctional epithelium in healthy subjects.^13^ Some investigators reported that up-regulation of CEACAM1 is associated with development of chronic immune-mediated diseases, such as psoriasis and lichen planus, and also chronic inflammatory diseases such as Crohn’s disease^14 , 31 , 32^. Furthermore, proinflammatory mediators and microorganisms in various cell lines has been shown to induce CEACAM1 expression. In other studies, proinflammatory cytokines, such as IFN-γ^31^ increased CEACAM1 expression in skin keratinocytes and in epithelial and endothelial cells, following stimulation with bacterial pathogens such as *Neisseria* .^9^ Klaile, et al.^33^ (2017), reported that live *C. albicans* increase CEACAM1 expression in intestinal epithelial cells. In this study, we found that heat-killed and live *C. albicans* cells increase CEACAM1 expression in oral keratinocytes. Therefore, *Candida* cell wall components can activate immune systems related to CEACAM1 expression in oral keratinocytes.

β-glucan, a cell-wall component of *Candida* recognized by the innate system, showed a capability to induce phagocytosis, cytotoxic activities, and pro-inflammatory cytokine production in macrophages.^34^ Although the induction of CEACAM1 expression by β-glucan has never been reported, this study found that β-GPs increase this expression in oral keratinocytes. On the other hand, Dectin-1 and C-type lectin receptors, characterized as major β-glucan receptors, express themselves in immune cells as human monocytes, macrophages, dendric cells, neutrophils, and eosinophils, as well as in non-immune cells, including human bronchial epithelial and intestinal epithelial cells.^35^ Furthermore, the latter study showed Dectin-1 functions as a trigger for effective immune response by recognition of β-glucan. We previously found that Dectin-1 was expressed in oral keratinocytes, whereas knockdown of Dectin-1 had no effect on fungal β-GP-induced CEACAM1 expression in the present study (data not shown). Therefore, the regulatory mechanism related to CEACAM1 expression induced by β-GPs in oral keratinocytes is likely different from that induced by Dectin-1.

HO-1 is an enzyme known to degrade heme and generate carbon monoxide and biliverdin, while causing release of iron, which is stored within the iron-binding protein ferritin.^36^ Moreover, the enzyme is stress inducible and upregulated in response to cellular stress conditions, such as oxidative stress, caused by reactive oxygen species (ROS). ^29^ HO-1 has an important role in decreasing oxidant-induced injury during inflammatory processes, whereas the overexpression can promote resistance to apoptosis, induce cell proliferation, and alleviate inflammation.^37^ Another study found HO-1 controlling various bacterial and viral infections *in vitro* and *vivo* examinations, which contributed to the elucidation of the immunoregulatory mechanisms induced by microorganisms.^38^ In our previous study, we found that β-GP-induced HO-1 regulate host defense against stress caused by *Candida* infection in oral epithelium, and the present findings showed that knockdown of CEACAM1 resulted in decreased β-GPs-induced HO-1, suggesting that CEACAM1 in oral keratinocytes is crucial in the induction of HO-1 to protect against cellular stress during *Candida* infection.

ROS-mediated oxidative stress activates cell mechanisms by releasing multiple cell-death factors^39^. Furthermore, excessive ROS production activates Nrf2/antioxidant response element signaling for defense against intracellular oxidative stress and regulation of increased HO-1 expression, which provide cell protective effects including anti-apoptotic and anti-inflammatory functions.^30 , 40^ Our previous findings showed that β-GPs induced intercellular ROS production in RT7 within 1 h, while translocation of Nrf2 into the nucleus increased HO-1 expression. In the study, a specific siRNA for CEACAM1 inhibited intercellular ROS induction after exposure to β-GPs, suggesting that CEACAM1 acts upstream of ROS and translocation of Nrf2, and regulates HO-1 expression.

CEACAM1 mediates adhesion and internalization,^41^which is crucial for mucosal colonization by various bacterial pathogens. Several different microorganisms, including *Neisseria gonorrhoeae, Neisseria meningitidis, Escherichia coli, Moraxella catarrhalis, Haemophilus influenzae, Salmonella* species, and *Helicobacter pylori,* can bind to CEACAM1 via various surface proteins that are structurally unrelated, whereas the target of these pathogens are the specific extracellular immunoglobulin V-like (Ig V-like) amino-terminal domain of CEACAMs.^41^ Recently, Klaile reported direct binding of *C. albicans* to CEACAM1 in intestinal epithelial cells via Ig V-like domains.^33^ Those authors examined the effects of heat-killed *C. albicans* and the ones treated by Zymolyase to undergo deglycosylation on binding to recombinant CEACAM1 to determine CEACAM1 binding to *Candida* cell wall components. However, those treatments decreased the interaction of CEACAM1 and *C. albicans* . Based on previous reports and on the present study, we considered that *C. albicans* binds to CEACAM1 in oral keratinocytes during candida infection of oral epithelium, and that β-glucan exposed on surface of *C. albicans* can activate CEACAM1 expression. Therefore, β-glucan increases ROS-mediated signaling resulting in increased HO-1 expression in oral keratinocytes. Furthermore, CEACAM1 in oral keratinocytes may participate in host immune defense response against *C. albicans* infection.

## Conclusions

To our knowledge, this is the first study to demonstrate that β-GPs enhance CEACAM1 expression in oral keratinocytes. We found an association between CEACAM1 and β-GPs -induced HO-1 expression via ROS signaling. Therefore, CEACAM1 in oral keratinocytes may have a critical role in regulating HO-1 for host immune defense during *Candida* infection.
